# Successful breastfeeding following a level II NICU stay in Qatar – a longitudinal study

**DOI:** 10.1186/s13006-022-00513-5

**Published:** 2022-11-08

**Authors:** Brijroy Viswanathan, Rajai El Bedaywi, Ahmed Tomerak, Sarfrazul Abedin, Prem Chandra

**Affiliations:** 1 NICU, Al Wakra Hospital, Hamad Medical Corporation, Doha, Qatar; 2 Medical Research Department, Hamad Medical Corporation, Doha, Qatar

**Keywords:** Breastfeeding, Feeding practices after NICU discharge, Preterm feeding

## Abstract

**Background:**

Exclusive breastfeeding is an essential need for mothers and newborn babies, but cultural practices and employment demands significantly influence feeding practices. The association between neonatal intensive care unit (NICU) admission and breastfeeding outcomes are variable. Data for Qatar and Middle East, in particular, are limited. Hence, this study aims to estimate the rate of breastfeeding at the time of NICU discharge and the rate of successful breastfeeding after NICU discharge in Qatar during well-baby follow-ups.

**Methods:**

This quantitative longitudinal study was conducted over 18 months from January 2019 and included neonates born in Al Wakra Hospital admitted to the NICU. Demographic data, feeding during NICU stay and at discharge were obtained from lactation charts. Data regarding feeding practices after discharge were obtained through a questionnaire administered at 4 weeks and 8 weeks in well-baby clinics. Descriptive statistics and logistic regression analyses were performed to determine the rates of breastfeeding and the association between the various factors.

**Results:**

Of the 678 participants screened, 364 were eligible for analysis. The rates of exclusive breastfeeding were 20% (73/364), 54% (197/364) and 42% (153/364) at discharge, 4 weeks and 8 weeks, respectively. Any breastfeeding was 64% (233/364), 40% (146/364) and 43% (157/364) at discharge, 4 weeks and 8 weeks, respectively. Logistic regression analysis showed that neonates who had NICU stays longer than 4 days had a higher rate of exclusive breastfeeding at discharge (adjusted odds ratio 3.000; 95% CI 1.25, 7.198) but had a reduced rate of breastfeeding and higher rate of formula feeding during follow-ups. Although breastfeeding rates were better in preterm infants at NICU discharge, regression analysis showed that none of the other factors, including gestation and maternal education had a significant association with the rate of exclusive breastfeeding at the time of discharge or during follow-ups.

**Conclusions:**

The overall breastfeeding rates from this level II NICU in Qatar are better than previously available data. Studies with extended follow-up and assessment of intervention methods should be planned to improve and sustain the practice of exclusive breastfeeding.

**Supplementary Information:**

The online version contains supplementary material available at 10.1186/s13006-022-00513-5.

## Background

The benefits of exclusive breastfeeding have been well recognised and the World Health Organization (WHO) recommends it for the first 6 months of an infant’s life, followed by continued breastfeeding with gradual introduction of solid foods for up to 2 years [1–3]. According to the United Nations International Children’s Emergency Fund (UNICEF), the exclusive breastfeeding rate for zero- to five-month-old babies in Qatar when last updated in 2012 was 29%, compared to the global rate of 37% [4]. The decision to breastfeed is greatly influenced by breastfeeding knowledge and awareness of the potential benefits of breastfeeding, which may be affected by cultural practices and environmental factors [5]. Although preterm birth is a risk factor for early cessation of breastfeeding, conflicting evidence remains regarding the association between NICU admission and breastfeeding outcomes among preterm infants [6]. The importance of initiating direct breastfeeding during the NICU stay and its impact on the duration of prolonged breastfeeding was studied by Pined, who observed that mothers who initiated breast milk feeding but did not put their infants to the breast in the NICU were no longer providing breast milk for their infants at NICU discharge [7]. A few other similarly conducted studies also showed a decreasing trend of breastfeeding rates after NICU discharge, but such data are not available from Qatar (see Tables 6 and 7). Progress in female labour force participation in the Middle East has been slow and, despite advances in education, remains less than 30% according to World Bank figures released in 2012 [8]. Yet, data on the impact of such employment demands on breastfeeding from Qatar are limited. Qatar’s National Development Strategy (2011–16) emphasized the integration of early prevention and intervention for obesity and other non-communicable diseases into different aspects of the health care system with a special focus on improving maternal and child health [9]. Without a clear understanding of the cultural factors that influence breastfeeding attitudes and practices in the current socioeconomic context in Qatar between Arab women and the immigrant population, health care professionals’ ability to develop and implement programs to promote exclusive breastfeeding is likely to be limited [9]. We prospectively studied breastfeeding rates during a level II NICU stay in Qatar and the subsequent follow-up of these mothers after discharge from the NICU to determine their exclusive breastfeeding adherence, cultural influence, and impact of support from family and the work environment on breastfeeding.

## Methods

A quantitative, longitudinal descriptive method was employed in this study. The primary outcome variable was the estimation of the rate of exclusive breastfeeding and any breastfeeding (breast feed plus formula feed) at the time of NICU discharge and breastfeeding practices at 4 weeks and 8 weeks after NICU discharge. The secondary objective of the study was to examine the impact of various factors on breastfeeding practices, such as maternal and neonatal characteristics, culture, employment status and health education.

The study was conducted in the NICU and the well-baby clinics of Al Wakra Hospital. The NICU at Al Wakra Hospital is a level II NICU that caters to neonates born at more than 30 weeks and more than 1.2 kg at birth. There are approximately 5000–6000 deliveries and 1200–1500 NICU admissions per year. The total study duration was 18 months starting from 1 January 2019. During this time, we recruited the eligible participants and followed them in the well-baby clinics for 8 weeks. The inclusion criteria included all neonates born in Al Wakra Hospital and admitted to the NICU of Al Wakra Hospital since birth and continued until discharge irrespective of gestation or birth weight. Babies admitted to Al Wakra NICU after being born at a different hospital and babies born in Al Wakra Hospital and subsequently transferred to other centres for further management were excluded. Screening of NICU admissions and recruitment of participants were initially delayed due to a lack of skilled research staff. Study amendments were made to add more research team members for data collection, and the study period was extended to 18 months.

**Fig. 1:**
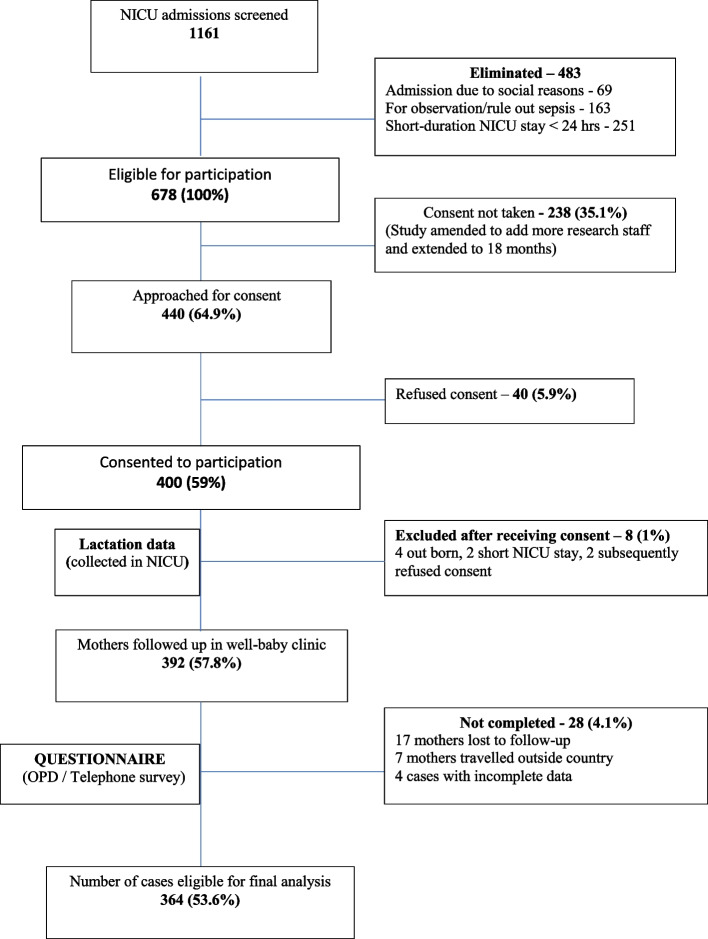
Study plan and patient flow

A schematic representation of the two-stage data collection method is shown in Fig. 1. In the first stage, during the NICU stay, a modified lactation chart (see Additional file 1) was used to collect data on infant characteristics such as gestational age, birth weight, primary diagnosis, type and time of initiation of feeding, length of stay, expressed milk feeding, direct breastfeeding and formula feeding details. These data were obtained from electronic medical records (CERNER) and from the feeding details of each newborn baby documented by the lactation nurse.

During the second stage, the babies discharged from the NICU were followed up in routine well-baby clinics. A validated questionnaire was used to collect data regarding the sociodemographic characteristics, maternal characteristics, obstetric factors and breastfeeding practices of mothers during the well-baby follow-up [10, 11]. Details of health education to promote breastfeeding, such as personnel, number of visits, and time and method of education, were also assessed through this questionnaire (see Additional file 2). These data were obtained at the first follow-up visit within 4 weeks of discharge and subsequently at the second follow-up within 8 weeks. The questionnaire was administered by a face-to-face interview in the well-baby clinic or by a telephone interview for those who failed to attend follow-up visits. Telephone interviews occurred mainly during the COVID-19 pandemic, when most of the outpatient department follow-up appointments were cancelled. Both lactation chart data and questionnaire data were entered into Excel independently and analysed using statistical methods. Exclusive breast milk feeding was defined as feeding directly from the breast or feeding with expressed breast milk only in the past 24 hrs before NICU discharge and all feeding after discharge. Any breastfeeding was identified when breast milk feeding was received at least once, and the rest of the feeding was infant formula feeding. Formula feeding only was identified when all feedings were given with infant formula only without any direct breastfeeding or expressed breast milk feeding [12, 13]. STROBE checklists were used for the design and reporting of the study.

### Ethical considerations

Ethical approval was obtained from the Institutional Review Board and Medical Research Department of Qatar (MRC -01-18-158). Participants were included in the study after obtaining signed consent, with permission for telephone interviews.

### Statistical analysis

The published breastfeeding rates for level II NICUs alone are limited, but the overall prevalence of exclusive breastfeeding at discharge among NICUs ranges between 20 and 40% [14, 15]. The sample size calculated (formula used: *n* = [DEFF*Np(1-p)] / [(d^2^ / Z^2^_1-α / 2_*(N-1) + p* (1-p)], where N: population size; p: the expected prevalence of the primary outcome estimates; d: the precision of the estimate; Zα: standard normal variate-the value of z from the standard probability tables; DEFF: design effect) with a 95% confidence interval for a prevalence of 30% and margin of error of 5% was 323 [16]. We recruited a total of 400 cases, assuming loss to follow-up as high as 15%. Descriptive statistics were used to summarize all demographic characteristics of the participants. Normally distributed data and results are reported as the mean and standard deviation (SD); the remaining results are reported as the median and range. Categorical variables are reported as frequencies and percentages. The proportions of exclusive breastfeeding, formula and any breastfeeding (breastfeeding plus infant formula feeding) for different age groups of infants were calculated, and the corresponding 95% confidence interval (CI) was computed to measure the precision of the prevalence estimate. Associations between two or more categorical variables were assessed using the Chi-square (χ2) test or Fisher’s exact test as appropriate. Adjusted odds ratios (AOR) were calculated by using logistic regression analysis to identify associations between exclusive breastfeeding vs. nonexclusive breastfeeding with various neonatal characteristics (gestation, NICU stay) and maternal parameters (age of mother, language, parity, method of delivery, educational status, occupation and family income). Logistic regression analysis was also performed between exclusive breastfeeding and nonexclusive breastfeeding to determine the association between breastfeeding education and breastfeeding support methods. All *P*-values presented are two-tailed, and *P*-values < 0.05 were considered statistically significant. All statistical analyses were performed using the statistical package SPSS 22.0 (SPSS Inc. Chicago, IL) software.

## Results

Of the 678 cases screened, 400 infants were recruited after obtaining informed consent. A total of 364 cases were eligible for complete data analysis after exclusion of eight infants and 28 mothers due to refusal of consent and loss to follow-up (Fig. 1). The baseline demographic details of the neonates and parents are presented in Table 1.

**Table 1:** Baseline data for neonates and mothers

Parameters	Results - number (%)
Gestation age weeks - Mean (SD)	37.33 (2.78), range: 27–41
• 27–31 + 6 weeks	19 (5.2%)
• 32–33 + 6	28 (7.7%)
• 34–36 + 6	57 (15.7%)
• > 37 weeks	260 (71.4%)
Weight kg - Mean (SD)	2.954 (0.795), range: 1.12–4.97
Sex	
▪ Male	210 (57.7%)
▪ Female	154 (43.3%)
Mode of delivery	
▪ Vaginal	177 (48.6%)
▪ LSCS	187 (51.4%)
NICU stay duration days – Median	3 days, range: 1–55
Parity	
• Primipara	146 (40.1%)
• Multipara	218 (59.9%)
Education status of mother	
▪ Secondary	71 (19.5%)
▪ College	217 (59.6%)
▪ University	72 (19.9%)
Occupation - mother	
• Not employed	251 (69%)
• Employed	113 (31%)
Family income / month	
▪ Less than QR.5000	39 (11.9%)
▪ QR.5000 – QR.15000	213 (65.13%)
▪ More than QR.15000	75 (22.9%)
Nationality	
• Qatari	14 (3.8%)
• Middle East including GCC	46 (12.6%)
• Indian subcontinent	187 (51.5%)
• Southeast Asia	36 (9.9%)
• Africa and other nationalities	81 (22.3)

*GCC* Gulf cooperation countries, *LSCS* Lower segment Cesarean section, *NICU* neonatal intensive care unit, *QR* Qatari riyal, *SD* standard deviation

### Feeding practices at discharge, at 4 weeks and at 8 weeks

**Fig. 2:**
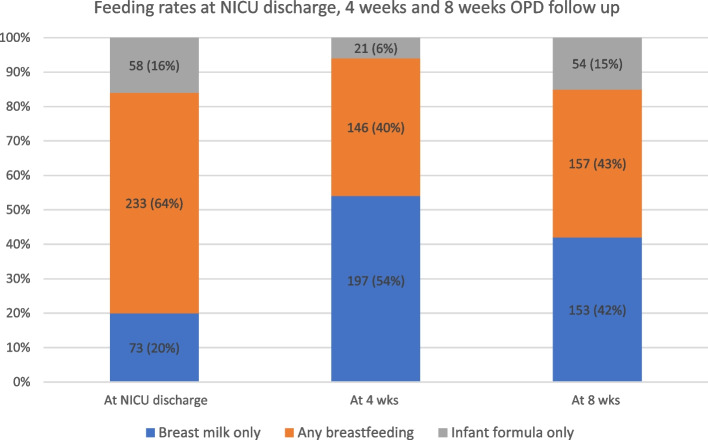
Feeding practices at discharge, 4-week follow-up and 8-week follow-up

Figure 2 shows the rates of exclusive breastfeeding, exclusive infant formula feeding and any breastfeeding at the time of NICU discharge, at 4 weeks and at 8 weeks. The rates of exclusive breastfeeding were 20% at discharge (73 / 364; 95% CI 16.2, 24.4), 54% at 4 weeks (197/364; 95% CI 48.9, 59.1) and 42% at 8 weeks (153 / 364; 95% CI 37, 47.1). The rates of any breastfeeding were 64% (233 / 364; 95% CI 58.9, 68.7), 40% (146 / 364; 95% CI 35.2, 45.2) and 43% (157 / 364; 95% CI 38.1, 48.2) at discharge, 4weeks and 8 weeks, respectively. The rates of exclusive formula feeding were 16% (58 / 364; 95% CI 12.5, 20), 6% (21 / 364; 95% CI 3.8, 8.6) and 15% (54 / 364; 95% CI 11.5, 18.8) at discharge, 4 weeks and 8 weeks, respectively.

#### Feeding practices compared with neonatal and maternal parameters

The breastfeeding rates at discharge, 4 weeks and 8 weeks were compared with gestational age, duration of NICU stay, mode of delivery, parity, language, maternal educational level, occupational status, and family income. These data are shown in Table 2.

**Table 2:** Feeding practices and neonatal and maternal parameters

Parameters: (numbers)		Feeding practices at discharge, number (%)			Feeding practices at 4 weeks, number (%)			Feeding practices at 8 weeks, number (%)		
		Breast milk only	Infant formula only	Any breast milk	Breast milk only	Infant formula only	Any breast milk	Breast milk only	Infant formula only	Any breast milk
**Sex**	Male (210)	36 (17.1)	35 (16.7)	139 (66.2)	114 (54.3)	13 (6.2)	83 (39.5)	92 (43.8)	35 (16.7)	83 (39.5)
**Mode of delivery**	Vaginal (177)	34 (19.2)	16 (9)	127 (71.8)	100 (56.5)	10 (5.6)	67 (37.9)	79 (44.6)	26 (14.7)	72 (40.7)
	LSCS (187)	39 (20.9)	42 (22.5)	106 (56.7)	97 (51.9)	11 (5.9)	79 (42.2)	74 (39.6)	28 (15)	85 (45.5)
**Gestational age (weeks)**	27–31 (19)	14 (73.7)	1 (5.3)	4 (21.1)	7 (36.8)	1 (5.3)	11 (57.9)	4 (21.1)	4 (21.1)	11 (57.9)
	32–33 (28)	17 (60.7)	3 (10.7)	8 (26.8)	18 (64.3)	4 (14.3)	6 (21.4)	11 (39.3)	5 (17.9)	12 (42.9)
	34–36 (58)	10 (17.2)	9 (15.5)	39 (67.2)	28 (48.3)	0 (0)	30 (51.7)	22 (37.9)	8 (13.8)	28 (48.3)
	37–41 (259)	32, (12.4)	45 (17.4)	182 (70.3)	144 (55.6)	16 (6.2)	99 (38.2)	116 (44.8)	37 (14.3)	106 (40.9)
**NICU stay duration (days)**	1 to 3 days (188))	14 (7.4)	47 (25)	127 (67.6)	109 (58)	11 (5.9)	68 (36.2)	84 (44.7)	23 (12.2)	81 (43.1)
	4 to 7 days (110)	18 (16.4)	9 (8.2)	83 (75.5)	63 (57.3)	7 (6.4)	40 (36.4)	51 (46.4)	16 (14.5)	43 (39.1)
	8 to 14 days (29)	14 (48.3)	10 (3.4)	14 (48.3)	6 (20.7)	2 (6.9)	21 (72.4)	6 (20.7)	8 (27.6)	15 (51.7
	> 14 days (37)	27 (73)	1 (2.7)	9 (24.3)	19 (51.4)	1 (2.7)	17 (45.9)	12 (32.4)	7 (18.9)	18 (48.6)
**Parity**	Primipara (146)	30 (20.5)	17 (11.6)	99 (67.8)	70 (47.9	7 (4.8)	69 (47.3)	57 (39)	21 (14.4)	68 (46.6)
	Multipara (218)	43 (19.7)	41 (18.8)	134 (61.5)	127 (58.3)	14 (6.4)	77 (35.3)	96 (44)	33 (15.1)	89 (40.8)
**Language**	Arabic (130)	25 (19.2))	25 (19.2)	80 (61.5)	66 (50.8)	11 (8.5)	53 (40.8)	56 (43.1)	29 (22.3)	45 (34.6)
	Non-Arabic (234)	48 (20.5)	32 (13.7)	154 (65.8)	131 (56)	10 (4.3)	93 (39.7)	97 (41.5)	24 (10.3)	113 (48.3)
**Mother’s age**	< 25 year (52)	10 (19.2)	6 (11.5)	36 (69.2)	29 (55.8)	1 (1.9)	22 (42.3)	23 (44.2)	9 (17.3)	20 (38.5)
	25 to 35 (273)	53 (19.4)	43 (15.8)	177 (64.8)	154 (56.4)	15 (5.5)	104 (38.1)	121 (44.3)	36 (13.2)	116 (42.5)
	> 35 year (39)	10 (25.6)	9 (23.1)	20 (51.3)	14 (35.9)	5 (12.8)	20 (51.3)	9 (23.1)	9 (23.1)	21 (53.8)
**Mother’s education**	Secondary (71)	11 (15.5)	13 (18.3)	47 (66.2)	32 (45.1)	7 (9.9)	32 (45.1)	25 (35.2)	18 (25.4)	28 (39.4)
	College (217)	45 (20.7)	37 (17.1)	135 (62.2)	124 (57.1)	12 (5.5)	81 (37.3)	97 (44.7)	29 (13.4)	91 (41.9)
	University (72)	16 (22.2)	7 (9.7)	49 (68.1)	40 (55.6)	2 (2.8)	30 (41.7)	30 (41.7)	7 (9.7)	35 (48.6)
**Mother’s occupation**	Not employed (251)	48 (19.1)	44 (17.5)	159 (63.3)	142 (56.6)	13 (5.2)	96 (38.2)	118 (47)	36 (14.3)	97 (38.6)
	Employed (113)	25 (22.1)	14 (12.4)	74 (65.5)	55 (48.7)	8 (7.1)	50 (44.2)	35 (31)	18 (15.9)	60 (53.1)
**Family Income**	QR. < 5 k (39)	6 (15.3)	9 (23)	24 (61.5)	17 (43.5)	5 (12.8)	17 (43.5)	16 (41)	11 (28.2)	12 (30.7)
	QR. 5 to 15 k (213)	41 (19.2)	36 (16.9)	136 (63.8)	128 (60)	11 (5.1)	74 (34.7)	102 (47.8)	28 (13.1)	83 (38.9)
	QR. > 15 (75)	14 (18.6)	11 (14.6)	50 (66.6)	33 (44)	5 (6.6)	37 (49.4)	25 (33.4)	12 (16)	38 (50.6)

*K* thousands*, LSCS* lower segment Cesarean section, *NICU* neonatal intensive care unit, *QR* Qatari riyal, *US* United States dollar

5000 QR = USD 1375;15,000 QR = USD 4125

Term gestation predominated in the study population, and the median was 38 weeks. When comparing different gestational age groups and the breastfeeding type and rate of feeding, it was observed that neonates who had a lower gestational age had better breastfeeding rates at the time of discharge (exclusive breastfeeding for preterm birth < 34 weeks was 66% (31 / 47) and for gestation > 35 weeks was 13% (42 / 317)), but multivariable analysis did not show a statistically significant association between the gestational age and mode of feeding at the time of NICU discharge or during follow-up at 4 weeks and 8 weeks (Table 3).

**Table 3:** Adjusted analysis for exclusive breastfeeding vs. nonexclusive breastfeeding at discharge, 4 weeks and 8 weeks, with various neonatal and maternal parameters

Newborn and maternal parameters	Exclusive breastfeeding at discharge	Exclusive breastfeeding at 4 weeks	Exclusive breastfeeding at 8 weeks
	Adjusted odds ratio (95% CI)	Adjusted odds ratio (95% CI)	Adjusted odds ratio (95% CI)
^**a**^**Gestation (32–33 + 6 wk)**	1.50 (0.28, 7.88)	5.09 (0.98, 26.27)	4.26 (0.83, 21.83)
**Gestation (34–36 + 6 wk)**	0.50 (0.09, 2.91)	1.34 (0.25, 7.09)	2.59 (0.47, 14.25)
**Gestation > 37 wk)**	0.58 (0.10, 3.45)	1.28 (0.24, 6.90)	3.33 (0.60, 18.42)
^**b**^**NICU stay (4–7 days)**	3.00 (1.25, 7.20)	0.70 (0.40, 1.22)	0.87 (0.50, 1.51)
**NICU stay (8–14 days)**	11.68 (3.20, 42.69)	0.18 (0.05, 0.64)	0.46 (0.14, 1.51)
**NICU stay (> 15 days)**	30.65 (6.62, 141.89)	0.59 (0.15, 2.33)	1.07 (0.30, 3.86)
^**c**^**Language (non-Arabic)**	0.71 (0.21, 2.42)	1.42 (0.62, 3.26)	1.28 (0.56, 2.91)
^**d**^**Age of mother (25–35 yr)**	0.68 (0.23, 2.00)	0.79 (0.37, 1.69)	0.98 (0.47, 2.04)
**Age of mother (> 35 yr)**	0.74 (0.17, 3.19)	0.25 (0.08, 0.74)	0.35 (0.11, 1.07)
^**e**^**Parity (multipara)**	0.97 (0.45, 2.08)	1.99 (1.18, 3.37)	1.43 (0.85, 2.40)
^**f**^**Mode of delivery (LSCS)**	0.88 (0.43, 1.80)	0.95 (0.58, 1.53)	0.75 (0.46, 1.21)
^**g**^**Education mother (college)**	1.73 (0.58, 5.11)	1.38 (0.69, 2.75)	1.56 (0.78, 3.11)
**Education mother (postgraduate)**	2.45 (0.69, 8.77)	1.50 (0.65, 3.47)	1.68 (0.72, 3.89)
^**h**^**Occupation mother (**employed**)**	1.28 (0.61, 2.7)	0.73 (0.43, 1.21)	0.51 (0.29, 0.87)
^**i**^**Income (5–15 k QR)**	0.99 (0.29, 3.39)	2.21 (0.98, 4.99)	1.73 (0.76, 3.93)
**Income (> 15 k QR)**	0.78 (0.18, 3.24)	1.19 (0.47, 3.06)	1.07 (0.41, 2.79)

*K* thousands*, LSCS* lower segment Cesarean section, *NICU* neonatal intensive care unit, *QR* Qatari riyal, *USD* United States dollar

^a^Reference group: 27–31 + 6 weeks gestation, ^b^Reference group: group 1–3 days NICU stay, ^c^Reference group: Arabic speaking, ^d^Reference group: mother’s age < 25 years, ^e^Reference group: primiparous mothers, ^f^Reference group: vaginal delivery, ^g^Reference group: secondary (school) education group, ^h^Reference group: non employed mothers, ^i^Reference group: income < 5000 QR group; 5000 QR = USD 1375;15,000 QR = USD 4125

The duration of the NICU stay had a wide range from one to 55 days, with a median of 3 days. When comparing breastfeeding type and rates with the duration of NICU stay, neonates who had NICU stays less than 4 days had exclusive breastfeeding rates of only 7.4% (14 / 188) (Table 2). Adjusted analysis showed higher exclusive breastfeeding rates at the time of NICU discharge for neonates with NICU stays longer than 1–3 days. Adjusted odds of exclusive breastfeeding for NICU stays of 4–7 days were 3.000 (95% CI 1.25, 7.198) compared with the reference group of NICU stays of 1–3 days; NICU stays of 8–14 days had an AOR of 11.679 (95% CI 3.19, 42.69); NICU stays > 15 days had AOR 30.648 (95% CI 6.62, 141.89). However, this association between feeding types and the duration of the NICU stay was no longer significant at 8 weeks (Table 3).

When compared with other neonatal and maternal parameters by multivariable regression analysis, there was no significant relation between the age of parents, educational status of mothers or income group and the method of feeding at NICU discharge or during the four-week and eight -week follow-ups. However, employed mothers were found to have lower exclusive breastfeeding at 8 weeks (AOR 0.51; 95% CI 0.29, 0.87).

Neonates delivered by lower segment cesarean section (LSCS) were noted to have higher infant formula feeding rates at the time of NICU discharge (23%; 42 / 187 vs. 9%; 16 / 177), but multivariable regression analysis did not show a statistically significant association (Tables 2 and 3). Similarly, Arabic-speaking mothers were noted to have higher exclusive infant formula feeding practices at the eight-week follow-up (22%; 29 / 130 vs. 10%; 24 / 234), but this observation was not statistically significant by multivariable regression analysis.

#### Feeding practices in relation to breastfeeding education and support

Feeding education and its relationship with the mode of feeding are summarized in Table 4.

**Table 4:** Feeding practices and breastfeeding education and support

Parameters:	Numbers (%)	Feeding practices at discharge, number (%)			Feeding practices at 4 weeks, number (%)			Feeding practices at 8 weeks, number (%)		
		Breast milk only	Infant formula only	Any breast milk	Breast milk only	Infant formula only	Any breast milk	Breast milk only	Infant formula only	Any breast milk
**BF advice done**	347 / 364 (95.3)	70 / 73 (95.8)	56 / 58 (96)	221 / 233 (95)	188 / 197 (95)	20 / 21 (95)	139 / 146 (95)	145 / 153 (95)	51 / 54 (94)	151 / 157 (96)
**Advised by doctor**	242 / 364 (66.4)	47 / 73 (64.3)	34 / 58 (59)	161 / 233 (69)	127 / 197 (64)	14 / 21 (67)	101 / 146 (69)	93/153 (61)	37 / 54 (68)	112 / 157 (71)
**Advised by nurse**	327 / 364 (89.8)	68 / 73 (93.1)	52 / 58 (90)	207 / 233 (89)	178 / 197 (90)	18 / 21 (86)	131 / 146 (90)	135 / 153 (88)	45 / 54 (83)	147 / 157 (94)
**Timing of education**	Prepartum 167 / 347 (48)	30 / 70 (42.8)	29 / 56 (52)	108 / 221 (49	97 / 188 (51)	10 / 20 (50)	60 / 139 (43)	75 / 145 (52)	21 / 51 (41)	71 / 15 1 (47)
	Postpartum 70 / 347 (50)	35 / 70 (50)	26 / 56 (46)	109 / 221 (49)	84 / 188 (45)	9 / 20 (45)	77 / 139 (55)	67 / 145 (46)	30 / 51 (59)	73 / 151 (48)
	Both 10 / 347 (2.8)	5 / 70 (7.1)	1 / 56 (1.7)	4 / 221 (1.8)	7 / 188 (3.7)	1 / 20 (5)	2 / 139 (1.4)	3 / 145 (2)	0 / 51 (0)	7 / 151 (4.6)
**Mode of education**	Verbal 301 / 348 (86.4)	61 / 70 (87.7)	50 / 56 (89)	190 / 222 (85)	165 / 188 (88)	13 / 20 (65)	123 / 140 (88)	123 / 145 (85)	47 / 51 (92)	131 / 152 (86)
	Written & verbal 47 / 348 (13.5)	9 / 70 (12.8)	6 / 56 (10.6)	32 / 222 (14)	23 / 188 (12.2)	7 / 20 (35)	17 / 140 (12.1)	22 / 145 (15.4)	4 / 51 (7.8)	21 / 152 (13.6)
**Follow up education BF**	220 / 364 (60.4)	45 / 73 (61.6)	31 / 58 (53)	144 / 233 (62)	128 / 197 (65)	15 / 21 (71)	77 / 146 (53)	96 / 153 (63)	28 / 54 (52)	96 / 157 (61)
**BF support by family**	333 / 364 (91.5)	67 / 73 (91.7)	55 / 58 (95)	211 / 233 (90)	181 / 197 (92)	16 / 21 (76)	136 / 146 (93)	138 / 153 (90)	49 / 54 (91)	146 / 157 (93)
**BF support by friend**	72 / 364 (19.8)	16 / 73 (21.9)	10 / 58 (17)	46 / 233 (19.7)	46 / 197 (23.)	1 / 21 (4.7)	25 / 146 (17)	31 / 153 (20)	8 / 54 (15)	33 / 157 (21)
**BF support by staff**	222 / 364 (61)	46 / 73 (63)	27 / 58 (46)	149 / 233 (64)	119 / 197 (60)	10 / 21 (48)	93 / 146 (64)	86 / 153 (56)	26 / 54 (48)	110 / 157 (70)
**Visit from NICU to encourage BF**	183 / 364 (50)	40 / 73 (54.7)	31 / 58 (53)	112 / 233 (48)	95 / 197 (48)	12 / 21 (57)	76 / 146 (52)	67 / 153 (44)	28 / 54 (52)	88 / 157 (56)

*BF* breastfeeding, *NICU* neonatal intensive care unit

Ninety-five percent (347 / 364) of mothers said that they were advised and recommended to breastfeed, but only 48% (167 / 347) said that this education was given prepartum and 86.4% (301 / 348) said that it was given by a verbal method only. Ninety-one percent (331 / 364) of mothers said that they received family support for breastfeeding, 61% (222 / 364) said they received support from hospital staff, and 20% (72 / 364) said they received support from friends. We used logistic regression to calculate the AOR to find the association of exclusive breastfeeding with breastfeeding education and support. We did not find a statistically significant relationship between exclusive breastfeeding and the mode of education and support (odds ratios of various parameters are presented in Table 5).

**Table 5:** Adjusted analysis for exclusive breastfeeding vs. nonexclusive breastfeeding at discharge, 4 weeks and 8 weeks, for mode of education

Parameters:	Exclusive breastfeeding at discharge	Exclusive breastfeeding at 4 weeks	Exclusive breastfeeding at 8 weeks
	Adjusted odds ratio (95% CI)	Adjusted odds ratio (95% CI)	Adjusted odds ratio (95% CI)
^**a**^**BF education given antenatally**	1.06 (0.61, 1.85)	1.41 (0.90, 2.23)	1.2 (0.76, 1.91)
^**b**^**Breastfeeding education any time**	0.29 (0.03, 2.80)	1.5 (0.24, 9.40)	1.14 (0.18, 7.33)
^**c**^**BF education by doctor**	0.84 (0.42, 1.66)	0.66 (0.38, 1.17)	0.58 (0.33, 1.02)
^**d**^**BF education by nurse**	2.28 (0.48, 10.88)	1.21 (0.45, 3.29)	0.7 (0.26, 1.91)
**Mode of education** ^**e**^**Written & verbal**	1.07 (0.50, 2.28)	0.88 (0.48, 1.62)	1.38 (0.75, 2.56)
^**f**^**BF follow up education after initial counselling**	1 (0.56, 1.79)	0.65 (0.40, 1.06)	0.8 (0.49, 1.30)
^**g**^**BF support by family**	1.16 (0.41, 3.25)	1.26 (0.55, 2.86)	0.89 (0.38, 2.07)
^**h**^**BF support by friends**	1.11 (0.57, 2.16)	1.99 (1.12, 3.51)	1.35 (0.77, 2.37)
^**i**^**BF support by staff**	1.18 (0.58, 2.40)	1.09 (0.61, 1.96)	0.81 (0.45, 1.47)
^**j**^**BF education in PNW by NICU team**	1.35 (0.76, 2.40)	0.72 (0.45, 1.15)	0.5 (0.31, 0.81)

*BF* breastfeeding, *CI* confidence interval, *NICU* neonatal intensive care unit, *PNW* postnatal ward

^a^Reference group: “breast feeding (BF) education given postnatally”, ^b^Reference group: “no breastfeeding education received”, ^c^Reference group: “no BF education by doctor”, ^d^Reference group: “no BF education by nurses”, ^e^Reference group: “BF education by verbal method only”, ^f^Reference group: “no follow up BF education after initial counselling”, ^g^Reference group: “no BF support by family”, ^h^Reference group: “no BF support by friends”, ^i^Reference group: “no BF support by staff”, ^j^Reference group: “no BF education in PNW by NICU team staff”

### Breastfeeding attitudes

During the four-week follow-up, mothers were asked about their attitudes and beliefs about breastfeeding and their preferences for infant formula milk. Although 82% (298 / 364) said that formula milk could lead to overfeeding, 16% (58 / 364) of mothers believed that formula milk was healthier. Whereas 33% (120 / 364) preferred formula milk for night-time feeding and for travel, 13% (46 / 364) believed that their baby’s crying may have been due to low breast milk and opted for formula milk feeding during that time. A total of 16.5% (60 / 363) of mothers felt that formula milk was better for employed mothers.

## Discussion

### Rates of breastfeeding

The key findings from this study (Fig. 2) are that the rate of exclusive breast milk feeding at the time of NICU discharge was 20% and the rate of any breastfeeding (breastfeeding + formula feeding) was 64%. A post-discharge follow-up survey at 4 weeks and 8 weeks showed that the rates of exclusive breastfeeding were 54 and 42%, respectively, and any breastfeeding rates were 40 and 43%, respectively. The exclusive formula feeding rates were 16, 6 and 15% at discharge, at 4 weeks and at 8 weeks, respectively. Similar NICU post-discharge data from Qatar have not been previously published. The few studies from Qatar and the Middle East that examined breastfeeding during the first 6 months of life found a range between 18 and 68% (Table 6) [17–22].

**Table 6:** Summary of a few studies with exclusive breastfeeding rates from Qatar and the Middle East

Study	Exclusive breastfeeding (Assessment time)	Method	Sample size	Location
Kayyali and Al-tawil [17]	32% (Birth – 12 months)	Questionnaire	340/well baby	Qatar / well- baby clinic
Al-Kohji et al. [18]	18.9% (< 6 months)	Questionnaire	Arab mothers	Qatar PHCC
Hendaus et al. [19]	24.3% (< 6 months)	Telephone interview	453 / well baby	Qatar / HMC
Alzaheb [20]	20.5% (< 6 months)	Meta-analysis of 19 studies		Middle East (9 countries)
Radwan et al. [21]	26.7% (6 months)	Questionnaire	374 / well baby	UAE
Al Tajir et al. [22]	48% (1 month) 13% (6 months)	Survey	221 / mothers	Sharjah / UAE

*HMC* Hamad Medical Corporation, *PHCC* Primary Health Care Corporation, *UAE* United Arab Emirates

### Breastfeeding rates in preterm neonates and association with NICU stay

In our study, 73% of neonates < 32 weeks had exclusive breast milk feeding at the time of NICU discharge, but this rate dropped to 37% at 4 weeks and 21% at 8 weeks. Although multivariable analysis did not show a statistically significant association between gestational age and the mode of feeding, it showed significantly better breastfeeding rates among neonates who stayed in the NICU for longer durations (Table 3). Better exclusive breastfeeding rates observed in the groups with longer NICU stays and very preterm neonates might indicate the support, education and motivation offered by the NICU staff to lactating mothers during the NICU stay and at the time of discharge. Other studies have reported a decreasing trend in exclusive breastfeeding rates after NICU discharge (Table 7) [12, 23–28]. In our study, the median gestational age was 38 weeks, so most of the babies were term and had short NICU stays. The predominant NICU admissions were due to transient tachypnoea of newborn in term babies delivered by LSCS with a short NICU stay, which might explain the low exclusive breastfeeding rates in this gestational age group at discharge. However, multivariable analysis did not find a significant association between exclusive breastfeeding groups and nonexclusive breastfeeding groups when compared with the mode of delivery (Table 3). After investigating the impact of intrapartum analgesia on infant feeding at hospital discharge, Jordan et al. [29] observed that intrapartum fentanyl at higher doses may impede the establishment of breastfeeding. Through a large obstetric dataset evaluation, Jordan et al. [30], showed that intrapartum medications and anesthetic methods have a negative impact on breastfeeding outcomes, which might contribute to the reduced breastfeeding rates at discharge following LSCS. We found that neonates who were born at term or late preterm who did not breastfeed at the time of NICU discharge could begin breastfeeding after NICU discharge. During follow-up, the breastfeeding rates showed improvement at 4 weeks as the neonates who had shorter NICU stays improved their breastfeeding rates (7.4 to 58%). However, very preterm neonates who had high breastfeeding rates at the time of NICU discharge started to show decreasing breastfeeding trends by 4 weeks (73 to 51%). By 8 weeks, the breastfeeding rates dropped at all gestational ages, but the association was not statistically significant (Table 3). These observations are similar to the published study data from NICUs across the world (Table 7). Niela-Vilén et al. performed a randomized controlled study of the breastfeeding experiences of preterm mothers in a social media peer group during and after NICU discharge and reported that preterm mothers expressed difficulty maintaining breast milk feeding after NICU discharge and that NICU nurse support and encouragement for breast pumping were needed [15].

**Table 7:** Summary of a few studies with NICU post-discharge breastfeeding rates

Briere et al. [23]	48% (At NICU discharge) 51% (1-month post-discharge) 26% (4 months corrected age)	Direct breastfeeding / at NICU discharge and follow up	46 / Preterm < 32 weeks	USA
Jiang and Jiang [24]	19% (1 month) 17% (3 months) 10% (6 months)	NICU post-discharge follow up	500 / preterm	Shanghai / China
Powers et al. [25]	49.7% (NICU at discharge)	NICU data	42,891 / 124 NICUs	USA
Balaminut et al. [26]	31% (1 month) 9% (6 months)	Hospital discharge / interview and medical records	84 / Preterm	Brazil
Maastrup et al. [12]	68% (at discharge) 13% (6 months)	NICU & post-discharge (Questionnaire)	1488 / preterm	Denmark
Kuan et al. [27]	59% (4 weeks) 47% (8 weeks)	Telephone interview	522 / women	USA
Ericson et al. [28]	64% (moderate preterm) and decreasing trend in all gestation	Neonatal quality register	29,455 / preterm	Sweden

*NICU* neonatal intensive care unit, *USA* United States of America

### Feeding practices in relation to maternal characteristics, feeding education and support

In our study, there was no significant relationship between the age of parents, parity or educational status of mothers and the feeding type and rates of breastfeeding at discharge or during the four-week and eight -week follow-ups. Although occupational status and income levels did not have an impact on breastfeeding at the time of NICU discharge, middle-income groups had better breastfeeding rates during follow-up, and employed mothers had higher exclusive formula feeding practices by 8 weeks (69% vs. 52%). Multivariable analysis also showed a statistically significant association between employed and non-employed mothers at 8 weeks: employed mothers were found to have less exclusive breastfeeding (AOR 0.51; 95% CI 0.29, 0.87). UNICEF data and other published data show a significant relation between the level of education and income levels with feeding practices [31]. When comparing Arabic-speaking and non-Arabic-speaking mothers, Arabic-speaking mothers had a higher rate of exclusive infant formula feeding at 8 weeks (22% vs. 10%). However, multivariable regression analysis showed that this observation was not significant when exclusive breastfeeding was compared with non-exclusive breastfeeding at 8 weeks between the two language groups (Table 3). Al-Kohji et al. made similar observations (higher formula feeding) among Arabic mothers in a 2009 study [18].

We did not find a statistically significant relationship between exclusive breastfeeding and the mode of education and support (odds ratios of various parameters are presented in Table 5), although 86% of patients reported that breastfeeding education was given only verbally and only 48% received such education and support prepartum. We also found that 33% of mothers preferred formula milk for night-time feeding and for feeding during travel. Based on a systematic literature review, Haroon et al. found that breastfeeding education and / or support increased exclusive breastfeeding rates and decreased the rate of no breastfeeding at birth, < 1 month and 1–5 months [32]. Chapman et al.’s systematic review concluded that breastfeeding peer committee initiatives are effective and can be scaled up in both developed and developing countries as part of well-coordinated national breastfeeding promotion or maternal-child health programs [33]. The WHO advocates that formal breastfeeding education should be provided over and above the breastfeeding information given as part of standard antenatal care and may include individual or group education sessions led by peer counsellors or health professionals, lactation consultants, the distribution of written materials, video demonstrations and the inclusion of prospective fathers in learning activities [34]. The small sample size in our study and the suboptimal education strategies undermine our findings related to feeding practices and breastfeeding education that are not in accordance with previously published reports.

### Strengths and limitations

This study shows reliable data regarding breastfeeding rates at the time of NICU discharge from a level II NICU in Qatar collected objectively by the health care team, although the follow-up survey information is self-reported data. A study amendment was made to add more research staff for data collection, resulting in a need for an extension of the study period and delay in data collection. This study showed that preterm neonates admitted to the NICU had a high percentage of exclusive breastfeeding at the time of discharge that declined after discharge, suggesting the need for continued breastfeeding support and motivation during follow-up. The study highlights quality improvement areas in the NICU for improving breastfeeding, such as motivating mothers during the antenatal period and initiating breastfeeding efforts for term babies in the NICU delivered by LSCS who may be discharged early without the opportunity to breastfeed during the NICU stay. Although this study population has varying nationalities, it is not a true representation of the country as the study was conducted in a level II NICU that caters to select areas of the country. The limited follow-up of 8 weeks substantially impacted the true assessment of feeding practices among the population. The observed decreasing trend in breastfeeding rates during the follow-up period was not statistically significant, and extended follow-up data and a larger sample size would provide a clearer understanding. A time series analysis would have helped to determine trends over time, although this was not the primary aim of this study. However, when we explored the testing assumptions, these assumptions did not meet our current research study data. Although the study examined the method of delivery and its impact on breastfeeding rates, we did not collect data regarding the medicines used during labour or their impact on breastfeeding rates. Much of the follow-up data were collected via telephone interviews due to the COVID-19 pandemic, and many mothers could not be interviewed in person. The study did not examine the method of breast milk expression, such as breast pumps, the utilization of kangaroo mother care or its impact on the duration of breastfeeding during follow-up.

## Conclusions

The overall breastfeeding rates from this single-level II NICU from Qatar are better than those from previously available data. Preterm neonates who had better breastfeeding rates at the time of NICU discharge yet subsequently declined during the outpatient department follow-up, indicate the impact of NICU nurse support and motivation, which was also demonstrated in many other studies. The tendency to add infant formula feeding to breastfeeding from the second month onwards, often due to the feeling of a reduced amount of breast milk, is concerning. Quality improvement strategies need to be planned and implemented to motivate mothers from the antenatal period and to continue to support them even after NICU discharge through exclusive breastfeeding clinics by NICU nurses and peer groups. Further studies with extended follow-up and assessment of intervention methods are suggested to better understand the findings of this study.

## Supplementary Information

**Additional file 1.** Lactation chart.

**Additional file 2.** Questionnaire.

## Data Availability

The datasets generated and / or analysed during the current study are not publicly available due to privacy protection and ethical obligations but are available (in de-identified form) from the corresponding author on reasonable request.

## Abbreviations

BF: Breastfeeding
CI: Confidence interval
COVID-19: Coronavirus disease 2019
DEFF: Design effect
GCC: Gulf cooperation council countries
HMC: Hamad Medical Corporation
K: Thousands
LSCS: Lower segment Cesarean section
NICU: Neonatal intensive care unit
PHCC: Primary Health Care Corporation
PNW: Postnatal ward
QR: Qatar riyal
SD: Standard deviation
UNICEF: United Nations International Children’s Emergency Fund
USD: United States dollar
WHO: World Health Organization
