# Supplementary material for: Successful breastfeeding following a level II NICU stay in Qatar – a longitudinal study
Source: Int Breastfeed J. 2022 Nov 8;17:76. doi: 10.1186/s13006-022-00513-5 (PMC9640813; doi:10.1186/s13006-022-00513-5)
Supplement: Supplementary file 1 — Additional file 1. Lactation chart. [file 13006_2022_513_MOESM1_ESM.pdf]

Serial number:

Lactation chart

Date of admission	
Gestation	
Birth weight	
Sex	
Diagnosis at admission	

Day of starting of feeding	
Type of feed at starting	
Days kept NPO after starting feeding	
TPN days	
Duration of tube feeding	
Days of assisted ventilation	

Date of discharge	
Gestation at discharge	
Feeding at discharge	BF/ EBM/ Formula / Both
Number of Feeding by (Direct BF) breast milk at discharge	
Diagnosis at discharge	

Average number of days receiving breast milk (EBM) feeding	
Average number of days receiving formula feeds	

Health education to mother	Verbal / written
Language of education	Mother tongue / English

Institutional Review Board Hamad Medical Corporation - Doha - Qatar Hamad

MRC-01-18-158 Validity: 16 06 2021 - 09 09 2021 E-stamped 21 Jun 2021

Age	Number of feeds by direct breast feeding	Number of feeds by EBM	Number of feeds by formula	TPN
day 1				
day 2				
day 3				
day 4				
day 5				
day 6				
day 7				
day 8				
day 9				
day 10				
day 11				
day 12				
day 13				
day 14				
day 15				
day 16				
day 17				
day 18				
day 19				
day 20				
day 21				
day 22				
day 23				
day 24				
day 25				
day 26				
day 27				
day 28				
day 29				
day 30				

Institutional Review Board Hamad Medical Corporation - Doha - Qatar
حمد
Hamad
MRC-01-18-158 Validity: 16 06 2021 - 09 09 2021 E-stamped 21 Jun 2021
