# Supplementary material for: Successful breastfeeding following a level II NICU stay in Qatar – a longitudinal study
Source: Int Breastfeed J. 2022 Nov 8;17:76. doi: 10.1186/s13006-022-00513-5 (PMC9640813; doi:10.1186/s13006-022-00513-5)
Supplement: Supplementary file 2 — Additional file 2. Questionnaire. [file 13006_2022_513_MOESM2_ESM.pdf]

Serial number: Date of interview:

Questionnaire

Socio Demography

Age of mother	
Age of father	
Age of baby	
Nationality	
Educational level (Mother)	Primary / secondary / college / postgraduate
Occupation (M)	House wife / part time / full time
Religion	Muslim / Christian / Others

Marital status	Married / Divorced / others
Educational level (Father)	Primary / secondary / college / postgraduate
Occupation (F)	
Income level	< 5 k / 5-10 / 10- 15 / 15- 20 / > 20
Address in Qatar	
Family support	Maternal / Paternal
Comments	

Maternal history

Gravida	Primi / multi
Medical illness	Cardiac/ renal / neuro / GIT/ others
Pregnancy related	PIH / GDD / Thyroid/ others
Ante natal care	Yes / No
Comments	

Obstetric

Mode of delivery	Vaginal / LSCS / instrumental
Complications of delivery	
Comments	

MRC-01-18-158 Validity: 17 10 2018 - 16 10 2019 E-stamped 30 October 2018

Breast-feeding education

Did you receive any advice regarding breast feeding	Yes / NO
If YES	
Who educated you regarding breast feeding	Doctor/ Nurse / others
Where did you get first breast feeding education	Antenatal clinic / delivery room/ post natal / NICU
When did you first receive information regarding breast feeding	Before delivery / after delivery
How did you receive information regarding breast feeding	Verbal / written
Did you understand the importance and concepts of breast feeding	Yes/ no / partially
Did you receive any follow up education regarding breast feeding	Yes / no
How many times, health care provider visit you to tell you about importance and need for initiating and continuing breast feeding	Once/ twice / 3 or more / don't remember
Which are the places were you received information regarding breast feed	Clinic / Labour room / PNW / NICU / don't know
Who all give you Support / encouragement for breast feeding	Family / friends/ staff
Did anyone visit you, while your baby was admitted to NICU and encouraged you to express breast milk and recommended to visit NICU at the earliest and prepare for breast feeding	Yes/ no<br>Comments:
Comments	

Breast-feeding practice

How do you feed at home	Exclusive breast feeding	Exclusively bottle	Both

If exclusively breast-fed

What made you decide to do exclusive breast feed	Education from hospital/ family belief / personal preference /
Do you practice expression of breast milk at home?	Yes / No
If yes<br>Which all situations you do expression of milk<br>How do you store it	

Institutional Review Board Hamad Medical Corporation Doha - Qatar Hamad
MRC-01-18-158 Validity: 17 10 2018 - 16 10 2019 E-stamped 30 October 2018

Who encouraged you to express breast milk	
Comments	

If giving both breast milk and formula,

Number of feeds by breast milk per day	

If fed with milk other than breast milk

What do you give	
How is it given?	Nipple / Cup / Spoon
Do you give anything other than milk such as water or tea or herbals	Yes / No
Why did you consider or started formula feeding	Not enough breast milk / baby was separated at birth/ lack of family support / due to my friends and relatives / its better for baby/ employment / others
Comments	
Did you receive help from staff during your visits to NICU for breast-feeding especially to help with positioning, latching and burping?	Yes / No
Did you receive help and advices from NICU staff at the time of discharge regarding breast-feeding at home?	Yes/ No

What advice was given at the time of discharge regarding feeding at home?	
Encouraged to breast feeding<br>Encouraged to express breast-feeding<br>Encouraged to give formula feeding<br>Encouraged to do all	

How often are you advised to feed at home?	
Whenever baby demands<br>Every 2 hours<br>Every 3 hours	
Comments	

Institutional Review Board Hamad Medical Corporation - Doha - Qatar
Hamad

MRC-01-18-158 Validity: 17 10 2018 - 16 10 2019 E-stamped 30 October 2018

Have you been given any advice regarding whom to contact for help, if you have problems with feeding your baby after you return home?	Yes/ No

If yes

What advice did you receive	To come to hospital / to call hotline number / To contact support group / others
Comments	

Have you ever been given any leaflets promoting breast milk substitutes from the hospital	Yes / No
If Yes<br>Specify<br>Leaflet of medical company with names of formula milk<br>Prescriptions for formula milk<br>Sample / bottle/ Pacifier<br>Others -	

Are you employed	Yes / no
If yes	

Do you receive any considerations at work place for lactation	Yes / No
If Yes<br>Specify<br>Extended leave for breast feeding<br>Permission between working hours for breast feeding<br>Facilities and support for expression and storage of breast milk	
Comments	

MRC-01-18-158 Validity: 17 10 2018 - 16 10 2019 E-stamped 30 October 2018

Attitudes and practices

Breast feeding is beneficial to mother and baby	Yes / No
Breast milk is the ideal and best milk for baby	Yes/ No
Do you think that babies crying always mean insufficient breast milk	Yes/ No
Do you think that formula fed babies are healthier than breast fed babies	Yes/ No
Do you know that formula fed babies are likely to be overfed than breast fed	Yes / No
Do you think that formula feeding is the best option for working mothers	Yes / No
Do you feel that working mothers should be given permission and extended leaves for exclusive breast feeding	Yes / No
Which milk do you prefer during travel	Breast milk / formula feed
Do you feel that public spaces should offer place and privacy for breast feeding	Yes/ No
Do you feel that promotions and advertisements by milk formula companies influence your decision about feeding the baby	Yes/ No
Did you feel pressure from relatives to resort to formula feeding	Yes/ No
Do you feel that your mother or mother in law helps in exclusive breast feeding	Yes/ No
Do you read health information provided in leaflets from clinics	Yes / No
You prefer written or verbal education regarding breast feeding and its benefits	Written/ verbal / both / others - specify
Prefer to formula feed during the night times	Yes/ No
What benefits do you know about breast feeding	----------------
Comments	

Institutional Review Board Hamad Medical Corporation · Doha - Qatar

MRC-01-18-158 Validity: 17 10 2018 - 16 10 2019 E-stamped 30 October 2018

Follow up at or before 8 weeks.

How do you feed at home	Exclusive breast feeding	Exclusively bottle	Both

If exclusively breast-fed

What made you decide to do exclusive breast feed	Education from hospital/ family belief / personal preference /
Do you practice expression of breast milk at home?	Yes / No
Comments	

If giving both breast milk and formula,

Number of feeds by breast milk per day	
Do you given anything other than milk feeds- either formula or human milk	Complementary feed / water/ tea / herbals / Nil

Are you employed	Yes / no
If yes	
Do you receive any considerations at work place for lactation	Yes / No
If Yes<br>Specify<br>Extended leave for breast feeding<br>Permission between working hours for breast feeding<br>Facilities and support for expression and storage of breast milk	
Comments	

Do you receive support at home	Yes/ No
Who supports at home if yes	Husband / mother/ in law/ servant/ relatives
Do you feel that working mothers should be given permission and extended leaves for exclusive breast feeding	Yes / No
Which milk do you prefer during travel	Breast milk / formula feed
Comments	

Institutional Review Board
Hamad Medical Corporation · Doha · Qatar
Hamad
